# A Dream: Perhaps a Delusion

**Published:** 1891-02-07

**Authors:** 


### The Doctor's Armchair.

### A DREAM: PERHAPS A DELUSION.

The consciousness of the medical world has been stirred and excited by the Koch controversy, as the spirits of a *bon vivant* are stimulated by champagne, or as the intellect of a Scotchman is roused by a metaphysical discussion. The ghosts of old theories have come forth from the shades, and have shown themselves to a generation that was almost unmindful of their existence. "For every disease there is a remedy, if we could but find it." That is the dream of the enthusiast who believes that this would be the best of all possible worlds, if man only were not "vile." We will not pause here to discuss the general question of human vileness; although we cannot pass this point in the road without expressing the conviction that theologians of all classes, and at all periods of time, have done their very utmost to "vilify" their kind. If those same theologians would condescend to give a little time to the study of natural science, and more particularly, if they would go to the school of Mr. Darwin for a brief period, they would probably offer us a juster and much more credible account of the situation.

The theory that for every disease there is a remedy, if we could only find it, is as pleasing as it is plausible. It labours under the doubt-inspiring fact, however, that it is of *à priori* origin. So many speculative hypotheses of this kind have been scattered to the winds by the labours of the inductionists, that one is sometimes tempted to take up the unphilosophical position that, whatever rests solely upon an *à priori* basis must necessarily be false. That would of course be absurd. But is it the case, as a matter of fact, that for every disease there is a remedy, if it could only be found? Before answering this question, we must turn the ambiguous word "remedy" into something more precise. If by "remedy" is meant "cure," then an exceedingly strong position may be taken up against the hypothesis; and the position is this—that up to the present time experience has not furnished us with a single remedy which can properly be called a "specific cure" for a "specific disease." Now, this is clearly a fundamental and also a staggering fact for those who believe in the doctrine that a remedy for every disease under the sun will be found if only we look carefully enough for it. If the world is not very old, neither is it very young; and if past experience has not furnished us with one single substance which definitely and certainly cures any one certain disease, there is a strong presumption that such substances will be as difficult to find in the future as they have been in the past. Solomon had a reputation for wisdom; and with the Jews "wisdom" often meant "worldly wisdom," or what the north countryman prefers to call "shrewdness." Solomon said: "That which hath been is that which shall be; and there is nothing new under the sun." Of course that is only true in a sense, but it is a very deep sense. We have our railways, it is true; but Solomon might have had them, too, if a Watt or a Stephenson had happened to be born in his reign. So also we have our steamships, our telegraphs, and our telephones. But the Egyptian civilisation, which was so long anterior to Solomon, might conceivably have had all these. They are but adaptations of forces which lay as ready to the hand of the cave-man as they now lie to the hand of an Edison or a Thomson.

There is a deep philosophical objection to the theory of specific cures for specific diseases. Of course, we do not here use the word "specific" in the medical sense, but in the philosophical. The objection is, that the giving of one particular substance for the cure of one particular disease savours of legerdemain; there is a suspicion of conjuring and trickery about it. We have shown that it is contrary to experience; but it is also contrary to analogy. "Short cuts" to any end are always to be suspected. For example, the half-educated theologian always gives you a formula whereby he guarantees you will find "salvation." "Do this," says he, or "believe that, and you will find both happiness and virtue." But, as a matter of experience, many people, though they both do and believe what they are told, find neither happiness nor virtue. The truth is, that virtuous life is not a series of juggling tricks with words or with ecclesiastical performances; it is the manful fight of a resolute soul against all mean and unworthy things, carried on from the dawn of reason to the day of death. Hitherto it has been the same with medicine. The physician has resources garnered by past ages; he has knowledge acquired by painful experiment in modern laboratories; he has the trained hand and eye; he has the sympathetic soul. Armed with these, he goes from bedside to bedside to wrestle in each case with the forces of disease, and, if possible, to trample death under his feet. He cannot bid the spectre depart by a wave of the hand, or a muttered incantation, or the injection of a subtle fluid into the life-blood of the sick man. His work is not so simple as that; at any rate, as yet. Will it ever be? The question descends into the deepest depths of the divine purpose in the creation of man, of the reason of suffering and pain, of the destiny that hinges upon human discipline, and of a hundred other difficulties upon which we cannot now enter. The theory of human existence that begins with a "chemical arrangement," that thinks all things possible to a "chemical modification," and that ends all by a "chemical transformation," may give hope of curing by conjuring, and of cheating death by a trick. But, in our judgment, that theory is as shallow and as blind and as insufficient as the schoolboy intellect can make it.

Is there, then, any just cause or impediment why Koch and Pasteur, Picq and Bertin, and any others who may so desire, should not experiment by the injection methods with which we have all been made so familiar of late? None whatever, that we know of. But there is every reason why such experimentors should have a philosophical conception of the nature of man, and of his physiological history. It is for them, as for other men of science, to remember that "art is long" whilst "life is short." The injection method, Jenner notwithstanding, is not merely in its infantile, but in its embryonic stage—embryonic in every sense of the word. What it will develop into—whether into a curious monstrosity, or into a perfect and serviceable form of art—it is impossible to foretell, any more than it is possible to foretell the final shape and ultimate life history of any other embryo that has but just entered upon its new and strange existence.

Experiment we know is the final test of the value of every method of medical treatment of every kind: and, therefore, to experiment, as to a scientific Cæsar, let the appeal be made. But then it is necessary that even the experiment be made with intelligence. For if it succeed, we want to know why it succeeds; and if it fail, we want to know why it fails. Mere experiment, unilluminated by intelligence, is almost as fatal to scientific progress as the *à priori* methods of the pre-Baconians. Why is the progress of science so exceedingly slow? It is because the scientists themselves, though so industrious in experimentation, are so dull in comprehending the true nature and the full significance of their own results. What have we witnessed during the last twenty years? One scientist after another travelling eternally round and round in the same circle, everlastingly threshing the very same straw that has been empty of corn for a generation. Bacon compelled modern scientists to pursue the methods of experiment. It is an infinite pity that he did not provide them with some means of gaining that noble intelligence which alone can make experiment fruitful. If Koch has done nothing else, he has at least done this—he has made us see that physical science divorced from the philosophic mind is just as fruitless as the philosophic mind divorced from physical science.

